# Rotigotine Objectively Improves Sleep in Parkinson's Disease: An Open-Label Pilot Study with Actigraphic Recording

**DOI:** 10.1155/2016/3724148

**Published:** 2016-02-14

**Authors:** Giovanna Calandra-Buonaura, Pietro Guaraldi, Andrea Doria, Stefano Zanigni, Stefania Nassetti, Valentina Favoni, Sabina Cevoli, Federica Provini, Pietro Cortelli

**Affiliations:** ^1^IRCCS Institute of Neurological Sciences of Bologna, Bellaria Hospital, 40139 Bologna, Italy; ^2^Department of Biomedical and Neuromotor Sciences, University of Bologna, 40123 Bologna, Italy; ^3^Neurology Outpatient Clinic, Department of Primary Care, Local Health Authority of Modena, Modena, Italy; ^4^Functional MR Unit, Policlinico S. Orsola-Malpighi, 40138 Bologna, Italy

## Abstract

Sleep disturbances represent important predictors of poor quality of life (QoL) in Parkinson's disease (PD). This open-label pilot study aimed to objectively assess, by means of actigraphic recording, effect of rotigotine on sleep in PD patients with self-reported sleep complaints. 15 PD patients underwent one-week actigraphic recording before (T0) and during (T1) rotigotine treatment, which was titrated to the dose subjectively improving motor symptoms (4–8 mg/24 h). Sleep disturbances, daytime sleepiness, cognitive performance, QoL, and depression were also evaluated with questionnaires. Actigraphic recordings showed a significant reduction in nocturnal motor activity and mean duration of wake episodes after sleep onset during rotigotine treatment compared to baseline. In 10 patients presenting objective evidence of poor sleep quality at T0 (sleep efficiency ≤ 85%), rotigotine also significantly improved other sleep parameters and further reduced nocturnal motor activity and mean duration of wake episodes. A significant decrease in number and duration of daytime sleep episodes was also observed at T1. Finally we confirmed that rotigotine significantly improves perceived sleep quality and QoL. Our study showed for the first time that rotigotine is associated with an objective improvement of nocturnal and diurnal sleep disturbances in PD patients with self-reported sleep complaints. This study is registered with AIFA-observational study registry number 12021.

## 1. Introduction

Sleep disturbances represent independent and important predictors of poor quality of life (QoL) in Parkinson's disease (PD) [1, 2].

Reduced sleep efficiency, arousal, and sleep fragmentation may be caused by PD pathology, motor symptoms (e.g., akinesia, rigidity, and dystonia), autonomic symptoms (nocturia), or a coexisting sleep disorder (e.g., restless legs syndrome, RLS; REM sleep behavior disorder, RBD). In addition patients could complain of excessive daytime sleepiness (EDS), increased diurnal sleep episodes, or sudden sleep attacks that have been related to nocturnal sleep disturbances, PD pathology, or use of dopaminergic agents [3]. The latter on the other hand were also demonstrated to improve sleep in PD. Rotigotine, a dopamine agonist available as transdermal patch and providing 24 hours (h) of drug delivery, was shown to subjectively improve sleep disturbances in PD patients [4–6]. However, an objective evaluation of this effect is lacking.

Actigraphy is routinely used to evaluate the sleep-wake cycle for long periods, through a portable device usually worn on the wrist using an accelerometer to detect movement, which is sampled several times a second. The accelerometer generates a voltage during each movement, which is amplified and band-pass filtered (2-3 Hz). The resulting signal is compared with a reference signal to determine if it exceeds a threshold for quantification and storage. Computerized scoring algorithms then qualified epoch of time (i.e., 60 seconds) as wake or sleep by comparing the activity score expressed in counts for that epoch to a threshold of activity set by the users. The motor activity is also quantified and reported as mean or median value of activity per epoch (or per minute for epoch of 60 seconds) [7]. In PD patients actigraphy proved appropriate to assess sleep quality, well correlating with subjective sleep measurements [8].

This open-label pilot study aimed to evaluate rotigotine's effect on sleep in PD patients with self-reported sleep complaints, providing also objective measurements by means of actigraphic recording.

## 2. Material and Methods

### 2.1. Selection of Patients

Between April 2013 and June 2014 we recruited from the outpatient Movement Disorders Clinic of our department 15 consecutive PD patients [1] who reported sleep complaints and showed a PD Sleep Scale-2 (PDSS-2) score ≥ 10 [9]. Only patients with a Hoehn and Yahr (H&Y) Score ≤ 3 [10], free from any medication for PD or treated with immediate-release levodopa (LD) and/or with the monoamine oxidase inhibitor rasagiline or selegiline on a stable dose for at least 4 weeks, requiring rotigotine to improve motor symptoms, were eligible. Exclusion criteria to enter the study were as follows: (1) global cognitive decline defined by a Mini Mental State Exam (MMSE) score < 24 [11]; (2) orthostatic hypotension [12]; (3) diagnosis of obstructive sleep apnea syndrome by means of Berlin Questionnaire [13]; (4) any clinically severe medical or psychiatric disease that could have interfered with study results; (5) concomitant treatment with drugs impacting sleep with the exception of benzodiazepines or selective serotonin reuptake inhibitors at low dose and stable for at least 6 months prior to enrollment.

The study was approved by the local ethics committee (AUSL of Bologna) and performed in accordance with the ethical standards laid down in the 1964 Declaration of Helsinki and subsequent amendments. Patients gave written informed consent before entering the study.

### 2.2. Protocol

Within 1 week after the screening, patients underwent the baseline visit (T0) including the following: (1) history taking, neurological examination, and blood pressure measurement to exclude orthostatic hypotension [12]; (2) quantification of motor impairment and disease severity (Unified PD Rating Scale-part III (UPDRS-III) and H&Y Score) [14]; (3) evaluation of sleep disturbances and daytime sleepiness by means of PDSS-2, RLS criteria and RLS rating scale, RBD questionnaire, and Epworth Sleep Scale (ESS) [15–17]; (4) PD Questionnaire-39 (PDQ-39) and Beck Depression Inventory (BDI) to assess QoL and depression [18, 19]; (5) global cognitive evaluation through MMSE [11]; (6) one-week wrist actigraphic recording (Mini Motionlogger Actigraph Advanced; Ambulatory Monitoring Inc., New York, NY, USA) with concurrent subjective sleep diary compilation. The portable device was worn on the nondominant wrist or on the unaffected arm in patients with resting arm tremor. The Cole-Kripke algorithm (with rescore options) was used to score the actigraphic data (Action-W version 2.7) [20]. Actigraphic measures during the nocturnal period (from lights off to lights on as stated on the sleep diary) included the following: sleep latency to persistent sleep (SL, latency in min of the first continuous block of at least 20 min sleep from the marked “lights-off” time); total sleep time (TST, overall min of sleep during the nocturnal recording); sleep efficiency (SE, TST/total recording time in percentage); activity mean (ACmean, mean activity score in counts/min); activity median (ACmedian, median activity score in counts/min); activity index (ACindex, percentage of TST with activity score > 0); total duration of wake time after sleep onset (WASO); number of wake episodes (number of periods of contiguous wakefulness epochs of 60 seconds) and mean duration of wake episodes. For the diurnal period (the remaining time of the 24 hr cycle after the exclusion of the nocturnal period) number and duration of sleep episodes were calculated. The sleep diary outcomes were the number of good sleep nights/week, the subjective estimation of the number and duration of nocturnal awakenings and of diurnal sleep episodes.

At the end of the actigraphic recording patients received rotigotine 24 h transdermal patch, which was titrated over 1–4 weeks to the optimal dose to subjectively improve motor symptoms, starting at 2 mg/24 h with weekly increments of 2 mg/24 h up to a maximum of 8 mg/24 h. The final dose was maintained for 4 weeks. The occurrence of rotigotine related adverse events and the emergence of impulse control disorders were monitored with weekly phone calls and additional visits when required.

When adverse events occurred during the titration period, rotigotine dose was back-titrated to the previous tolerated dose for the maintenance period. At the end of the maintenance period patients underwent a new visit (T1) including the same assessment performed at T0.

### 2.3. Statistical Analysis

Rotigotine's effect on sleep disturbances was evaluated by comparing actigraphic data, sleep diary, and questionnaire results obtained at T1 and T0. Mean values of 7 days were calculated for each parameter for statistical analysis.

Questionnaires and scales on motor performance, QoL, depression, and global cognitive profile were also compared between T1 and T0.

As deviation from a normal distribution was found data were reported as medians and 25th–75th percentiles and comparisons performed by the Wilcoxon signed-rank test. Statistical significance was set at *p* ≤ 0.05.

## 3. Results

All the 15 enrolled PD patients (12 males, mean age ± SD 67 ± 9 years, disease duration 5 ± 3 years) completed the study. At T0, 1 patient was free from PD medication, 1 was treated with selegiline, and 13 were treated with LD (mean daily dose ± SD = 333 ± 162 mg), in combination with rasagiline in 4 patients (Supplementary Table 1 in Supplementary Material available online at http://dx.doi.org/10.1155/2016/3724148).

At T1, 12 patients were taking the maximal rotigotine dose of 8 mg/24 h. Patient 1 referred adequate improvement of motor symptoms at 6 mg/24 h. Two patients decreased the dose during the titration phase to 6 mg/24 h (patient 10) and 4 mg/24 h (patient 15) as they experienced adverse events at higher dose (compulsive sexual behavior and irritability, resp.).

At T1 compared to T0 significant improvements of motor performance (UPDRS-III) and self-reported sleep complaints (PDSS-2) were observed (Table 1). Single item analysis of PDSS-2 showed a significant improvement in 9 out of 15 items including those exploring quality of sleep, motor symptoms at night, pain, and nocturia (Supplementary Table 2).

Four patients (2, 3, 7, and 11) reported RLS at T0 and a significant decrease in RLS severity while on rotigotine treatment (RLS severity at T0 is 31, 29, 36, and 21 and at T1 is 21, 18, 29, and 0). Ten patients had a positive questionnaire for RBD. Four out of the 6 patients reporting at least one RBD episode/week at T0 referred a RBD frequency decrease at T1.

ESS score was globally unchanged after rotigotine treatment (Table 1).

Actigraphic and sleep diary data are reported in Tables 2 and 3. During the nocturnal period at T1, we observed a significant reduction in mean duration of wake episodes and ACmean. When considering only the ten patients showing pathological SE (≤85%) at T0, we also observed a significant improvement in SE and a significant reduction in WASO, mean duration of wake episodes, ACmean, and ACmedian at T1.

Nights of good sleep per week were significantly increased and number and duration of nocturnal awakenings significantly decreased under rotigotine according to patients' diaries.

During the diurnal period actigraphic recording showed a significant reduction in number and duration of sleep episodes at T1, while only reduction in the number of sleep episodes emerged from patients' diaries.

Finally MMSE score, H&Y Score, and BDI score were not significantly different at T1 while a significant improvement of QoL was observed (Table 1).

## 4. Discussion

In this open-label pilot study we demonstrated, by means of actigraphic recordings, that the introduction of rotigotine was objectively associated with a significant reduction in motor activity and mean duration of wake episodes during nocturnal sleep in PD patients with self-reported sleep complaints. In patients with pathological SE at baseline (≤85%) [21, 22], rotigotine also significantly improved SE and WASO and further reduced motor activity and mean duration of wake episodes during sleep.

In agreement with previous studies we also confirmed that rotigotine improves perceived sleep quality in PD patients [4–6] and is associated with amelioration of QoL.

According to our actigraphic and patients' reported results, rotigotine's effect on sleep disturbances may depend on several factors: (1) reduction in motor activity during sleep, which could be related to the need to move limbs due to restlessness or to painful posturing, which may cause awakening or arousal from sleep; (2) improvement of a coexisting sleep disorder like RLS; (3) improvement of nocturia and painful posturing as what emerged from PDSS-2. Rotigotine may also contribute to the reduction of the number of spontaneous or movement related arousal times during sleep [23], which could be increased in PD patients [3]. This may explain the subjective sleep quality improvement in patients presenting normal SE at T0 and T1.

Rotigotine was well tolerated in this study. Sleep attacks or worsening of daytime sleepiness was not observed under treatment, during which on the contrary we found a decrease in number and duration of daytime sleep episodes. This result is clinically significant only for the duration of sleep episodes while their number was already low before treatment with rotigotine.

The main limitation of the study is the lack of a placebo group which does not allow us to evaluate the presence and magnitude of a placebo effect. However our subjective data are in agreement with the previous randomized placebo-controlled study showing a subjective sleep improvement with rotigotine [4].

## 5. Conclusions

We demonstrated for the first time by means of objective measurements a positive effect of rotigotine on nocturnal and diurnal sleep disturbances in PD patients with self-reported sleep complaints. Larger randomized placebo-controlled studies with actigraphic or polysomnographic recordings are warranted to confirm these data and define the effect of rotigotine on sleep fragmentation and arousal from sleep.

## Supplementary Material

Supplementary Table 1 shows the demographic and clinical features of the 15 PD patients enrolled in the study.
Supplementary Table 2 compares single items' score of the PDSS-2 questionnaire during rotigotine treatment (T1) and baseline (T0). A significant improvement of 9 out of 15 items was observed during rotigotine.

## Figures and Tables

**Table 1:** Scale results before (T0) and during (T1) rotigotine treatment.

Scale	T0	T1	*p*
UPDRS-III^*∗*^	26 (18–41)	22 (11–39)	0.018
H&Y	2 (2-2)	2 (2-2)	0.083
MMSE	28 (27–30)	30 (27–30)	0.121
PDSS-2^*∗*^	20 (16–30)	9 (4–20)	0.001
ESS	5 (4–6)	2 (3–7)	0.937
PDQ-39^*∗*^	34 (19–55)	29 (18–42)	0.016
BDI	7 (3–12)	6 (5–13)	0.977

UPDRS-III = Unified Parkinson's Disease (PD) Rating Scale-part III; H&Y = Hoehn and Yahr Score; MMSE = Mini Mental State Examination; PDSS-2 = PD Sleep Scale-2; ESS = Epworth Sleep Scale; PDQ-39 = PD Questionnaire-39; BDI = Beck Depression Inventory. Data are expressed as medians and 25th–75th percentiles. *∗* = statistical significance *p* ≤ 0.05.

**Table 2:** Actigraphic and sleep diary results before (T0) and during (T1) rotigotine treatment.

	T0	T1	*p*
*Nocturnal actigraphic data*			
SL (min)	42 (15–65)	15 (8–40)	0.363
TST (min)	373 (321–424)	379 (298–399)	0.609
SE (%)	82 (64–87)	82 (71–88)	0.118
WASO (min)	122 (65–169)	83 (66–136)	0.069
N. of wake episodes	19 (11–21)	19 (16–26)	0.164
Mean duration of wake episodes (min)^*∗*^	9 (6–12)	7 (4–8)	0.002
ACmean (counts/min)^*∗*^	37 (26–51)	29 (24–41)	0.020
ACmedian (counts/min)	5 (0–27)	4 (0–16)	0.077
ACindex	53 (39–75)	54 (39–71)	0.349

*Nocturnal sleep diary data*			
N. of good nights/week^*∗*^	3 (0–4)	4 (3–6)	0.004
N. of wake episodes^*∗*^	1 (0–2)	0 (0-1)	0.003
Total duration of wake episodes (min)^*∗*^	29 (5–60)	0 (0–38)	0.002

*Diurnal actigraphic data*			
N. of sleep episodes^*∗*^	1 (1-2)	1 (1-1)	0.014
Total duration of sleep episodes (min)^*∗*^	70 (36–155)	57 (27–77)	0.011

*Diurnal sleep diary data*			
N. of sleep episodes^*∗*^	1 (0-1)	0 (0-1)	0.046
Total duration of sleep episodes (min)	51 (17–94)	26 (9–69)	0.249

SL = sleep latency; min = minutes; TST = total sleep time; SE = sleep efficiency; WASO = wake after sleep onset; N. = number; ACmean = activity mean score; ACmedian = activity median score; ACindex = activity index. Data are expressed as median (25th–75th percentiles). *∗* = statistical significance *p* ≤ 0.05.

**Table 3:** Nocturnal actigraphic results before (T0) and during (T1) rotigotine treatment in 10 patients with sleep efficiency ≤ 85% at T0.

Scale	T0	T1	*p*
SL (min)	47 (23–66)	24 (7–40)	0.241
TST (min)	343 (289–373)	330 (286–397)	0.508
SE (%)^*∗*^	68 (51–82)	73 (69–83)	0.017
WASO (min)^*∗*^	147 (114–215)	109 (81–152)	0.013
Number of wake episodes	20 (19–22)	21 (16–28)	0.646
Mean duration of wake episodes (min)^*∗*^	11 (8–14)	7 (5–9)	0.005
ACmean^*∗*^	47 (36–69)	35 (28–44)	0.005
ACmedian^*∗*^	11 (5–30)	8 (0–17)	0.028
ACindex	64 (50–79)	65 (43–73)	0.114

SL = sleep latency; min = minutes; TST = total sleep time; SE = sleep efficiency; WASO = wake after sleep onset; ACmean = activity mean score; ACmedian = activity median score; ACindex = activity index. Data are expressed as median (25th–75th percentiles). *∗* = *p* ≤ 0.05.
