# Supplementary material for: Rotigotine Objectively Improves Sleep in Parkinson's Disease: An Open-Label Pilot Study with Actigraphic Recording
Source: Parkinsons Dis. 2016 Feb 14;2016:3724148. doi: 10.1155/2016/3724148 (PMC4769773; doi:10.1155/2016/3724148)
Supplement: Supplementary file 1 — Supplementary Table 1 shows the demographic and clinical features of the 15 PD patients enrolled in the study. Supplementary Table 2 compares single items' score of the PDSS-2 questionnaire during rotigotine treatment (T1) and baseline (T0). A significant improvement of 9 out of 15 items was observed during rotigotine. [file 3724148.f1.doc]

**Supplementary Table 1 and Table 2**

**Rotigotine objectively improves sleep in Parkinson disease: an open-label pilot study with actigraphic recording**

Giovanna Calandra-Buonaura, Pietro Guaraldi, Andrea Doria, Stefano Zanigni, Stefania Nassetti, Valentina Favoni, Sabina Cevoli, Federica Provini, Pietro Cortelli

**Supplementary Table 1. Patients’ features at T0**

| **Patient** | **Age (F/M)** | **Disease**  **Duration (y)** | **PD treatment**  **at T0** | **Concomitant diseases** | **Other therapies** |
| --- | --- | --- | --- | --- | --- |
| 1 | 68 M | 6 | LD 400 mg  Rasagiline 1 mg | Hypertension  Hypotiroidism | CCB  Levothyroxine |
| 2 | 65 M | 3 | LD 400 mg | Hypertension | BB  CCB  ASA |
| 3 | 68 F | 2 | LD 200 mg | Hypotiroidism  DM II * | levothyroxine  OADs |
| 4 | 53 F | 5 | LD 200 mg  Rasagiline 1 mg |  |  |
| 5 | 78 F | 8 | LD 300 mg | Depression | BZD |
| 6 | 61 M | 1 | Selegiline 10 mg | Hypertension | ACEI  BB |
| 7 | 75 M | 2 | LD 200 mg | Hypertension  DM II * | BB  clopidogrel  OADs |
| 8 | 74 M | 7 | LD 375 mg  Rasagiline 1 mg | Hypertension | ACEI  BB  ASA |
| 9 | 68 M | 6 | LD 200 mg | Hypertension | BB  CCB  ARB |
| 10 | 76 M | 9 | LD 700 |  |  |
| 11 | 80 M | 3 | LD 300 mg |  |  |
| 12 | 59 M | 8 | LD 200 mg  Rasagiline 1 mg |  |  |
| 13 | 70 M | 3 | LD 250 mg | Hypertension | ACEI  CCB  ASA |
| 14 | 49 M | 6 | LD 600 mg |  |  |
| 15 | 62 M | 2 | - | Depression | SSRI |

F = female; M = male; y = years; PD = Parkinson’s disease; LD = levodopa; mg = milligram; CCB = calcium channel blockers; BB = beta blockers; ASA = acetylsalicylic acid; DM = diabetes mellitus type II; ACEI = angiotensin converting enzyme inhibitors; OADS = oral antidiabetic drugs; BDZ = benzodiazepines; ARB = angiotensin receptor blockers; SSRI = selective serotonin reuptake inhibitors.

* In patients with DM II peripheral polyneuropathy was excluded.

**Supplementary Table 2.** PDSS-2 single items’ score before (T0) and during rotigotine treatment (T1).

| **Item n.** | **Description** | **T0** | **T1** | **p** |
| --- | --- | --- | --- | --- |
| 1 | Poor sleep quality* | 2 (1-4) | 0 (0-1) | 0.008 |
| 2 | Difficulty falling asleep | 0 (0-2) | 0 (0-2) | 0.606 |
| 3 | Difficulty staying asleep* | 4 (2-4) | 2 (0-3) | 0.018 |
| 4 | Limb restlessness* | 0 (0-4) | 0 (0-2) | 0.047 |
| 5 | Urge to move limbs * | 0 (0-4) | 0 (0-1) | 0.026 |
| 6 | Distressing dreams* | 1 (0-2) | 0 (0-1) | 0.013 |
| 7 | Distressing hallucinations | 0 (0-0) | 0 (0-0) | 0.102 |
| 8 | Got up to pass urine* | 4 (2-4) | 2 (1-4) | 0.040 |
| 9 | Uncomfortable sensation due to immobility* | 3 (0-4) | 0 (0-2) | 0.011 |
| 10 | Pain in arms or legs | 1 (0-3) | 0 (0-2) | 0.301 |
| 11 | Muscle cramps in arms or legs | 0 (0-1) | 0 (0-0) | 0.131 |
| 12 | Painful posturing in the morning* | 0 (0-2) | 0 (0-0) | 0.034 |
| 13 | Tremor on waking | 0 (0-1) | 0 (0-1) | 0.234 |
| 14 | Sensation of sleepiness after waking in the morning* | 2 (0-4) | 1 (0-2) | 0.009 |
| 15 | Breathing problems/snoring | 0 (0-2) | 0 (0-0) | 0.140 |

Data are expressed as medians and 25th-75th percentiles.* = statistical significance p≤0.05
